# Accuracy of F-18 FDG PET/CT with optimal cut-offs of maximum standardized uptake value according to size for diagnosis of regional lymph node metastasis in patients with rectal cancer

**DOI:** 10.1186/s40644-018-0165-5

**Published:** 2018-09-14

**Authors:** Sung Uk Bae, Kyoung Sook Won, Bong-Il Song, Woon Kyung Jeong, Seong Kyu Baek, Hae Won Kim

**Affiliations:** 1 Department of Surgery, Keimyung University Dongsan Medical Center, Daegu, Republic of Korea; 2 Department of Nuclear Medicine, Keimyung University Dongsan Medical Center, 56 Dalseong-ro, Jung-gu, Daegu, 41931 Republic of Korea

**Keywords:** Rectal cancer, Lymph node metastasis, Maximum standardized uptake value, Partial volume effect, F-18 FDG, PET/CT

## Abstract

**Background:**

The low sensitivity of F-18 fluorodeoxyglucose (FDG) positron emission tomography/computed tomography (PET/CT) for the evaluation of metastatic lymph nodes (LNs) is mainly due to the partial volume effect in patients with rectal cancer. This retrospective study evaluated the diagnostic accuracy of F-18 FDG PET/CT with optimal cut-off values of the maximum standardized uptake value (SUV_max_), according to LN size, for the evaluation of regional LN in rectal cancer patients.

**Methods:**

This study included 176 patients with rectal cancer who underwent F-18 FDG PET/CT for initial staging. Patients were classified based on the long-axis diameter of the regional LN on CT images as small (≤ 7 mm; *n* = 118) and large (> 7 mm; *n* = 58) LN groups. The optimal cut-off value of SUV_max_ was determined for each group, using receiver operating characteristic curve analysis. Areas under the curve (AUC) were compared by C-statistics using two methods: the cut-off value of SUV_max_ optimized according to LN size, and a fixed SUV_max_ cut-off value of 2.5.

**Results:**

The optimal cut-off values of SUV_max_ for the small and large LN groups were 1.1, and 2.1, respectively. The sensitivity, specificity, and accuracy of F-18 FDG PET/CT using the optimal cut-off values were 90.6, 70.9, and 76.3% in the small LN group, and 68.6, 78.3, and 72.4% in the large LN group. The sensitivity, specificity, and accuracy of F-18 FDG PET/CT using the fixed cut-off value were 18.8, 100, and 78.0% in the small LN group, and 51.4, 87.0, and 65.5% in the large LN group. The AUC was significantly higher using the optimal cut-off values than the fixed cut-off value (0.808 vs. 0.594, *p* = 0.005) in the small LN group, but not in the large LN group (0.734 vs. 0.692, *p* = 0.429).

**Conclusions:**

Application of the lower cut-off value of SUV_max_ improves the diagnostic performance of F-18 FDG PET/CT for the evaluation of small regional LNs in patients with rectal cancer.

**Electronic supplementary material:**

The online version of this article (10.1186/s40644-018-0165-5) contains supplementary material, which is available to authorized users.

## Background

Globally, colorectal cancer is the second most common cancer in women and the third most common cancer in men [1]. In Korea, the rectum was the most common site of cancer among both men and women in 1999 and again in 2009 [2]. Lymph node (LN) metastasis is one of the most important prognostic factors for patients with rectal cancer [3]. Additionally, LN metastasis plays a primary role in the determination of the operability and the extent of LN dissection. Survival is directly related to the presence of residual metastatic LNs after the primary operation. The accurate diagnosis of LN metastasis in initial staging may improve the prognosis and allow the early use of second-line therapy in patients with rectal cancer [4].

Conventional computed tomography (CT) and magnetic resonance imaging (MRI) have been commonly used for LN staging in patients with rectal cancer. However, both CT and MRI are limited by low sensitivity in the evaluation of small metastatic LNs [5–8]. Recently, F-18 fluorodeoxyglucose (FDG) positron emission tomography/computed tomography (PET/CT) has been proven to be useful for the preoperative staging of rectal cancer by revealing metabolic information of the lesion [9–11]. However, F-18 FDG PET/CT has also shown low sensitivity for the detection of LN metastasis [12, 13]. The low sensitivity of F-18 FDG PET/CT in the evaluation of metastatic LNs is mainly due to the partial volume effect, which spills out of the radioactivity into the background of small lesions < 10 mm in size, leading to underestimation of the true standardized uptake value (SUV) [14–16].

Several methods have been developed to correct the partial volume effect, and have significantly improved the diagnostic accuracy of metastatic LNs [17, 18]. However, there have been several limitations of the clinical use of partial volume correction due to the complexity of the method. Any method to consider size differences of LNs on F-18 FDG PET/CT images must be practical. Previous studies of an F-18 FDG PET/CT quantitative approach used a fixed cut-off of the maximum standardized uptake value (SUV_max_) in the diagnosis of LN metastasis, without considering the size differences of the LNs. Application of optimal SUV_max_ cut-off values according to LN size may improve the sensitivity of F-18 FDG PET/CT and may be practically useful for evaluation of the regional LNs in patients with rectal cancer. Thus, the aim of this study was to evaluate the diagnostic accuracy of F-18 FDG PET/CT using optimal SUV_max_ cut-off values according to LN size to evaluate regional LNs in patients with rectal cancer.

## Subjects and methods

### Study population

We retrospectively analyzed the medical records of patients who underwent preoperative F-18 FDG PET/CT followed by curative operations for rectal cancer at our institution between January 2009 and August 2016. We excluded patients who underwent preoperative chemoradiation therapy and those with an interval of > 4 weeks between F-18 FDG PET/CT and surgery. A retrospective cross-sectional analysis was performed to review the surgical and pathological findings and the F-18 FDG PET/CT results. Patients were classified based on the long-axis diameter of the regional LN on CT images as small (≤ 7 mm; *n* = 118) and large (> 7 mm; *n* = 58) LN groups. The reference value for long-axis diameter was determined as 7 mm, because the partial volume effect is significant when the target of interest is smaller than 2 times of the PET/CT system’s full-width at half-maximum (FWHM) (< 8 mm) [18], and the long-axis diameter range on multiple detector CT has been reported as 7–10 mm for the diagnosis of metastatic regional LN [19, 20]. This study was approved by the Institutional Review Board of our institution.

### Histopathologic examination

All surgeries were performed by qualified, experienced colorectal surgeons. Mesorectal excisions were performed in all patients; extended LN dissections were only performed if metastatic LNs were detected in frozen biopsies. All resected LNs underwent histopathologic exams for pathologic confirmation while labeling the exact location. The sensitivity, specificity, and accuracy of F-18 FDG PET/CT were calculated using the histopathologic result as the gold standard. A true positive was defined as a match between the location of the metastatic LN on pathologic examination and the location of the positive LN on an F-18 FDG PET/CT image.

### F-18 FDG petPET/CT

Two different F-18 FDG PET/CT systems were used (Discovery STE 16, GE Healthcare, Milwaukee, WI, USA; and Biograph mCT 64, Siemens Healthcare, Knoxville, TN, USA). The patients were required to fast for > 6 h before the scan, and the blood glucose level was measured to confirm that the level was < 180 mg/dL before injecting the F-18 FDG. In patients with diabetes, administration of antihyperglycemic drugs was stopped 12 h before the scan. Patients received intravenous administration of 4.0 MBq/kg (Biograph mCT) and 7.0 MBq/kg (Discovery STE) F-18 FDG according to the PET/CT system. Patients were encouraged to rest during the F-18 FDG uptake period. Images were acquired 60 min after F-18 FDG administration. A non-contrast CT scan was obtained for attenuation correction and localization. Immediately after the CT scan, PET images were acquired from the base of the skull or top of the brain to the proximal thigh. The Discovery STE-16 PET/CT scanner acquired images with a slice thickness of 3.75 mm simultaneously for a longitudinal field of view (FOV) of 780 mm. The transaxial FOV was 70 cm, and the matrix size was 128 × 128. Spatial resolution in air was 4.29 mm FWHM. The PET images were reconstructed from CT data for attenuation correction using the OSEM iterative algorithm with 20 subsets and two iterations. The Biograph mCT-64 PET/CT scanner acquired images with a slice thickness of 3 mm simultaneously for a longitudinal FOV of 500 mm. The transaxial FOV was 58.8 cm, and the matrix size was 256 × 256. Spatial resolution in air was 4 mm FWHM. The PET images were reconstructed from CT data for attenuation correction using the TrueX algorithm and an all-pass filter with 21 subsets and two iterations.

An experienced nuclear physician blinded to the histopathologic and colonoscopic results reviewed the F-18 FDG PET/CT images on a workstation (Advantage Workstation version 4.3; GE Healthcare). The locations of the regional LNs were recorded as the perirectal, superior rectal, inferior mesenteric, or internal iliac areas. Suspicious lymph nodes less than 3 mm were ignored because they cannot be differentiated from vascular structures or other nonspecific soft tissue densities. The ROIs (long-axis diameter range, 3–17 mm) were drawn in consensus around the regional LNs, and the SUV_max_ was measured using each dedicated PET workstation (ADW version 4.3 for Discovery STE-16 and syngo MI for Biograph mCT-64). The optimal SUV_max_ cut-off values were determined using receiver operating characteristic curve (ROC) analysis for the small and large LN groups. When the measured SUV_max_ exceeded the optimal cut-off value or 2.5, the LN was considered positive. In addition, subgroup analyses were performed according to the PET/CT scanner (PET A and B), T stage (early and advanced T stages), and F-18 FDG uptake of the primary tumor (low and high tumor SUV_max_). Patients who were examined using the Discovery STE-16 PET/CT scanner were classified into the PET A group and patients who were examined using the Biograph mCT-64 PET/CT scanner were classified into the PET B group. Patients with T1 or T2 stage were classified into the early T stage group and patients with T3 or T4 stage were classified into the advanced T stage group. Patients with SUV_max_ of the primary tumor lower than 13.0, which was the median value of SUV_max_, were classified into the low tumor SUV_max_ group and patients with SUV_max_ of the primary tumor higher than 13.0 were classified into the high tumor SUV_max_ group. The optimal SUV_max_ cut-off values were determined for each subgroup.

### Statistical analyses

The optimal cut-off values of the SUV_max_ in each group and each subgroup were calculated using ROC analysis. The sensitivities, specificities, and accuracies of PET/CT using the optimal SUV_max_ cut-off values according to LN size, and a fixed SUV_max_ cut-off value of 2.5, were calculated for each group, each subgroup, and for all patients together. The areas under the curve (AUCs) of the optimal and fixed SUV_max_ cut-off values were compared using C-statistics. A *p*-value < 0.05 was considered significant.

## Results

### Patient characteristics

**Fig. 1:**
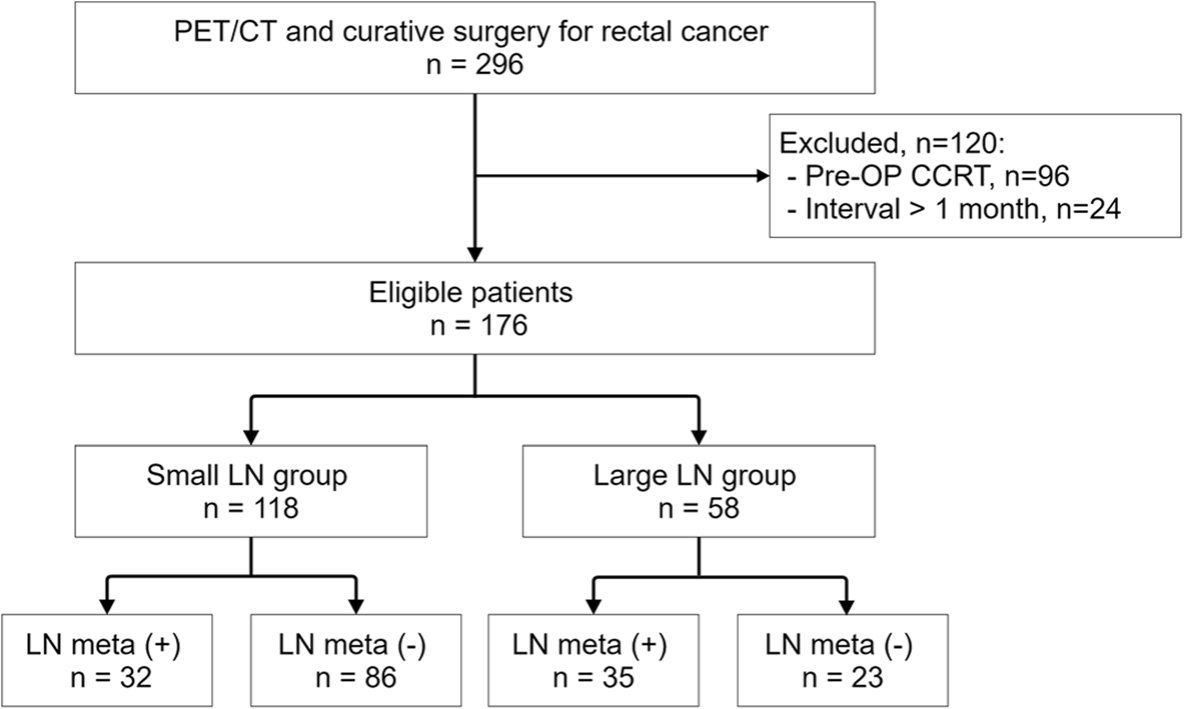
Patient STARD flow chart

Of 296 patients who underwent preoperative F-18 FDG PET/CT and follow-up curative surgery for rectal cancer, 120 patients were excluded from this study according to the exclusion criteria (Fig. 1). A total of 176 patients were included. Table 1 summarizes the patient characteristics. Patients were classified into the small (*n* = 118) or large (*n* = 58) LN groups. Regional LN metastasis was confirmed pathologically in 32 patients (27.1%) in the small LN group, and 35 patients (60.3%) in the large LN group.

**Table 1:** Patient characteristics

Characteristics^a^	Overall (*n* = 176)	Group^b^	
		Small LN^c^ (*n* = 118)	Large LN (*n* = 58)
Age, years	66.7 (10.4)	67.4 (9.3)	65.3 (12.4)
Male, %	56.8	61	48.3
AJCC^d^ Stage, *n*			
I	69	59	10
II	38	27	11
III	69	32	37
IV	0	0	0
LN diameter, mm	6.2 (3.0)	4.6 (1.7)	9.4 (2.5)
SUV_max_ of LN	1.8 (2.2)	1.2 (0.7)	3.2 (3.2)
PET/CT scanner, *n*			
Discovery STE-16	79	54	25
Biograph mCT-64	97	64	33
T stage, n			
T1–2	81	68	13
T3–4	95	50	45
SUV_max_ of primary tumor, n			
High SUV_max_ (< 13.0)	84	64	20
Low SUV_max_ (> 13.0)	92	54	38

^a^All values are presented as means (SD)

^b^Patients were categorized by the long-axis diameter of the regional LN, as follows: small LN, ≤ 7 mm; large LN, > 7 mm

^c^*LN* lymph node

^d^*AJCC* American Joint Committee on Cancer

The SUV_max_ of large LNs was significantly higher than that of small LNs in the overall patient analysis (3.2 vs. 1.2, *p* < 0.001). There was no significant difference in the SUV_max_ of small and large LNs between the PET A and B groups (1.2 vs. 1.2, *p* = 0.964 and 2.6 vs. 3.7, *p* = 210). The SUV_max_ of the small LN in the advanced T stage group was significantly higher than that in the early T stage group (1.5 vs. 1.0, *p* < 0.001), but the SUV_max_ of large LN was not significantly different between these two groups (3.6 vs. 2.0, *p* = 0.110). The SUV_max_ of the small LN in the high tumor SUV_max_ group was significantly higher than that in the low tumor SUV_max_ group (1.0 vs. 1.4, *p* < 0.016), but the SUV_max_ of large LN was not significantly different between these two groups (3.6 vs. 2.5, *p* = 0.230).

### Accuracy of F-18 FDG PET/CT

**Fig. 2:**
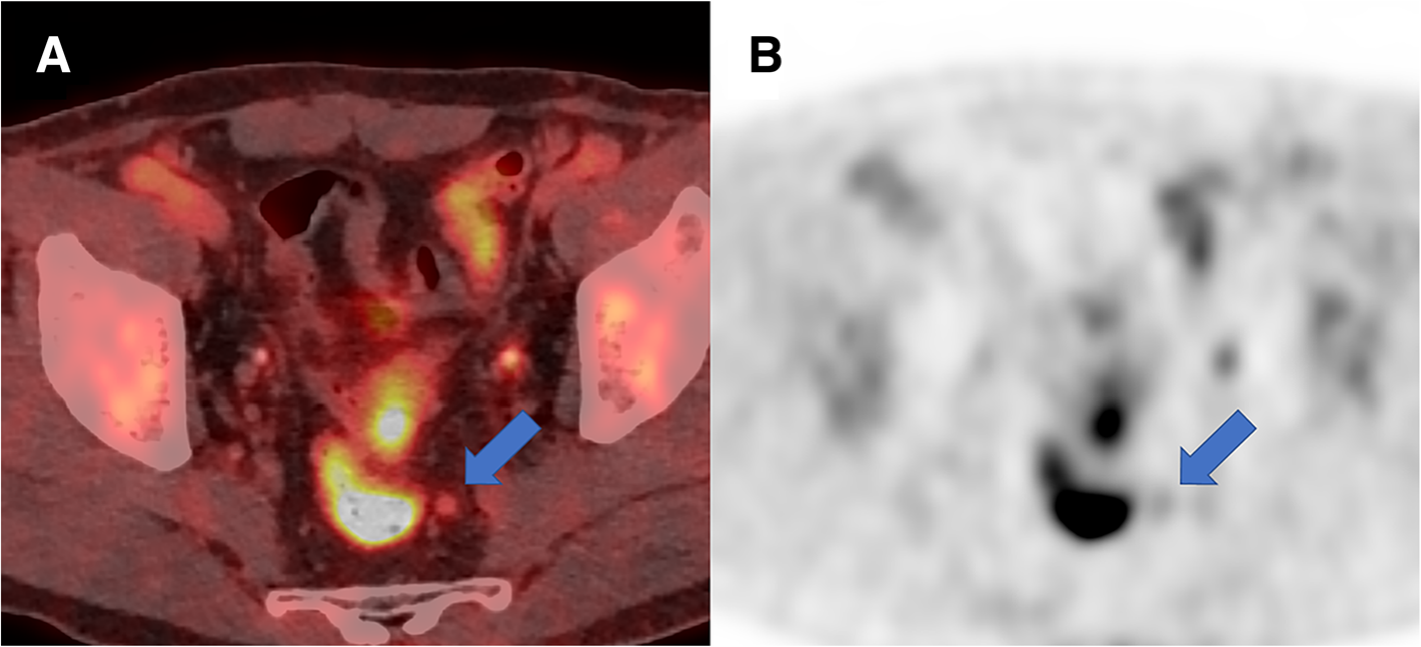
A representative case of regional LN metastasis predicted by optimal SUV_max_ cut-off values, but not by the fixed SUV_max_ cut-off value of 2.5. (**a**) A mildly hypermetabolic lymph node (arrow) was observed in the left perirectal region. The long-axis diameter of the LN was 6 mm, and the patient was classified into the small LN group according to the size criteria. (**b**) The SUV_max_ of the LN was 1.8, and exceeded the optimal SUV_max_ cut-off value of 1.1. Histopathologic examination revealed that the lesion was a metastatic LN

The optimal cut-off values of SUV_max_ for the diagnosis of regional LN metastasis were 1.1 in the small LN group, and 2.1 in the large LN group. The sensitivity, specificity, accuracy, and AUC using the optimal SUV_max_ cut-off values were 90.6, 70.9, 76.3%, and 0.808 in the small LN group, and 68.6, 78.3, 72.4%, and 0.734 in the large LN group, respectively (Table 2). Using the fixed SUV_max_ cut-off value of 2.5, the corresponding values were 18.8, 100, 78.0%, and 0.594 in the small LN group, and 51.4, 87.0, 65.5%, and 0.692 in the large LN group, respectively. The AUCs of PET/CT using the optimal cut-off values were significantly higher than those using the fixed cut-off value of 2.5 in the small LN group (*p* = 0.005). Figure 2 shows a representative case of regional LN metastasis that was predicted by using the optimal SUV_max_ cut-off values, but not by the fixed SUV_max_ cut-off value of 2.5. There was no significant difference in the AUC between the two methods in the large LN group (*p* = 0.429).

**Table 2:** Comparison of the diagnostic values between PET/CT using the cut-off values of SUV_max_ optimized according to the lymph node (LN) size and the fixed SUV_max_ cut-off value of 2.5

Group	Cut-off values	Sensitivity (%)	Specificity (%)	PPV^a^ (%)	NPV^b^ (%)	Accuracy (%)	AUC^c^	*p*
Overall	2.5	35.8	97.2	88.9	71.1	73.9	0.665	0.071
	Opt^d^	76.1	74.3	64.6	83.5	75	0.752	
Small LN	2.5	18.8	100	100	76.8	78	0.594	0.005
	1.1	90.6	70.9	53.7	95.3	76.3	0.808	
Large LN	2.5	51.4	87	85.7	54.1	65.5	0.692	0.429
	2.1	68.6	78.3	82.8	62.1	72.4	0.734	

^a^*PPV* positive predictive value

^b^*NPV* negative predictive value

^c^*AUC* area under the curve

^d^*Opt* optimal cut-off values of SUV_max_ (1.1 in the small LN group and 2.1 in the large LN group)

In overall patients, the sensitivity, specificity, accuracy, and AUC of F-18 FDG PET/CT, using the optimal cut-off values, were 76.1, 74.3, 75.0%, and 0.752, respectively whereas on using the fixed cut-off value, the sensitivity, specificity, accuracy, and AUC were 35.8, 97.2, 73.9%, and 0.665, respectively. The AUC of PET/CT using the optimal cut-off value was higher than that using the fixed cut-off value of 2.5 in all patients, but not statistically significant (*p* = 0.071).

Subgroup analysis was performed according to the PET/CT scanner. In the PET A group, the optimal cut-off values of SUV_max_ were 1.1 for small LN and 2.1 for large LN. In the PET B group, the optimal cut-off values of SUV_max_ were 1.0 for small LN and 1.9 for large LN. Table 3 shows the sensitivity, specificity, accuracy, and AUC in the PET A and B groups. In the small LN of the PET A group, the AUC using the optimal cut-off value was significantly higher than that using the fixed cut-off value of 2.5 (*p* = 0.047). There were no significant differences in the AUCs between PET/CT using the optimal and fixed cut-off values in the large LN of the PET A group (*p* = 0.866), as well as small and large LNs of the PET B group (*p* = 0.110 and *p* = 0.162). Subgroup analysis according to the T stage revealed that the optimal cut-off values were 0.9 for small LN and 1.8 for large LN in the early T stage group and 1.1 for small LN and 2.1 for large LN in the advanced T stage group. There were no significant differences in the AUCs between PET/CT using the optimal and fixed cut-off values in the small and large LNs of the early and advanced T stage groups (*p* = 0.188, *p* = 1.000, *p* = 0.231 and *p* = 0.822). Additional file 1: Table S1 shows the sensitivity, specificity, accuracy, and AUC in the early and advanced T stage groups. Subgroup analysis according to the SUV_max_ of the primary tumor revealed that the optimal cut-off values were 1.0 for small LN and 1.3 for large LN in the low tumor SUV_max_ group, and 1.1 for small LN and 2.1 for large LN in the high tumor SUV_max_ group. There were no significant differences in the AUCs between PET/CT using the optimal and fixed cut-off values in the small and large LNs of the low and high tumor SUV_max_ groups (*p* = 0.070, *p* = 0.908, *p* = 0.177 and *p* = 0.491). Additional file 2: Table S2 shows the sensitivity, specificity, accuracy, and AUC in the low and high tumor SUV_max_ groups.

**Table 3:** Comparison of diagnostic values between PET/CT using the optimized cut-off values and the fixed cut-off value of 2.5 in patients imaged by PET A and B

Groups	Cut-off values	Sensitivity (%)	Specificity (%)	PPV^a^ (%)	NPV^b^ (%)	Accuracy (%)	AUC^c^	*p*
PET A								
Overall	2.5	36.7	98.0	91.7	71.6	74.7	0.673	0.169
	Opt	76.7	77.6	67.6	84.4	77.2	0.771	
Small LN	2.5	18.8	100.0	100.0	74.5	75.9	0.594	0.047
	1.1	87.5	73.7	58.3	93.3	77.8	0.806	
Large LN	2.5	57.1	90.9	88.9	62.5	72.0	0.740	0.866
	2.1	78.6	72.7	78.6	72.7	76.0	0.756	
PET B								
Overall	2.5	35.1	96.7	86.7	70.7	73.2	0.659	0.520
	Opt	75.7	65.0	57.1	81.3	69.1	0.703	
Small LN	2.5	18.8	100.0	100.0	78.7	79.7	0.594	0.110
	1.0	93.8	60.4	44.1	96.7	68.8	0.771	
Large LN	2.5	47.6	83.3	83.3	47.6	60.6	0.655	0.162
	1.9	61.9	83.3	86.7	55.6	69.7	0.726	

^a^*PPV* positive predictive value

^b^*NPV* negative predictive value

^c^*AUC* area under the curve

^d^*Opt* optimal cut-off values of SUV_max_

## Discussion

The present study revealed improved diagnostic performance of F-18 FDG PET/CT in the evaluation of metastatic LNs in patients with rectal cancer using the optimal SUV_max_ cut-off values according to the size of the LN. Application of a lower SUV_max_ cut-off value to evaluate a small LN increased the sensitivity of PET/CT in the detection of metastatic LNs in patients with rectal cancer. The AUCs of the PET/CT with optimal SUV_max_ cut-off values were significantly higher than those with a fixed cut-off value of 2.5 in the small LN group. These results suggest that F-18 FDG PET/CT can diagnose LN metastasis as accurately in small LNs as in large LNs if a lower SUV_max_ cut-off value is applied. Although the concept of this hypothesis is widely known, the present study proved it practically in sufficient number of patients with rectal cancer.

LN metastasis in rectal cancer is directly correlated with prognosis. The 5-year survival rate is > 95% in rectal cancer patients without LN metastasis, but decreases to 50~ 70% in patients with LN metastasis [3]. Additionally, the LN stage of rectal cancer is one of the most important determining factors for adjuvant chemotherapy and extended LN dissection [21, 22]. The procedure of choice for rectal cancer patients with a clinical stage of N0 or N1 is total mesorectal excision, which is surgical excision of the mesorectal fat, including all LNs. In more advanced cancers with a clinical stage of N2, preoperative concurrent chemoradiotherapy is recommended. Extended LN dissection is required in patients with suspected metastatic LNs in the lateral pelvic region [23–25]. Application of the optimal SUV_max_ cut-off values according to the LN size allows determination of treatment strategies and improves the prognosis of patients with rectal cancer by improving the accuracy of the diagnosis of LN metastasis with F-18 FDG PET/CT.

F-18 FDG PET/CT is beneficial in the preoperative staging of rectal cancer, though it showed low sensitivity and accuracy in the diagnosis of LN metastasis [12, 13]. There is no definite evidence supporting F-18 FDG PET/CT as the routine clinical application in the evaluation of LN metastasis, though F-18 FDG PET/CT could be used to supplement the possibility of suspected metastatic LNs detected by other imaging modalities. For a quantitative approach to the diagnosis of LN metastasis on F-18 FDG PET/CT images, a fixed cut-off value of SUV_max_ of 2.5 has been commonly used to diagnose metastatic LNs [5, 12]. However, the sensitivity (38~ 65%) of F-18 FDG PET/CT in the diagnosis of LN metastasis were low compared to those of CT and MRI [13]. In accordance with previous studies, the corresponding values using a fixed SUV_max_ cut-off value of 2.5 in the present study were comparably low. However, there was significant improvement in the sensitivity of PET/CT when using an SUV_max_ cut-off value optimized according to LN size.

The primary cause of the low sensitivity of F-18 FDG PET/CT in the diagnosis of LN metastasis is the partial volume effect [14–16], which causes the underestimation of radioactivity concentration in structures with less than two to three times the spatial resolution of PET (4~ 5 mm). Due to the partial volume effect, the quantitative evaluation of LN metastasis has not been a routine practice in the interpretation of F-18 FDG PET/CT images. Due to the partial volume effect, lower optimal SUV_max_ cut-off values were determined for the evaluation of small LNs than for the large LNs. Several techniques have been developed to calibrate the partial volume effect. In addition, many studies revealed significant improvement in the diagnostic performance of F-18 FDG PET/CT for the determination of small lesions after partial volume correction [17, 18]. However, partial volume correction methods are generally too complex to be clinically applicable, and most require additional equipment or applications. Therefore, we applied the optimal SUV_max_ cut-off values according to LN size to compensate for the partial volume effect, which resulted in significant improvement in the AUC. This approach could be a more rapid and simpler method for calibration of the partial volume effect.

CT and MRI have conventionally been used to evaluate LN metastasis in rectal cancer by evaluating the size and shape of the LN [5, 6]. A diameter of 5~ 10 mm has been applied as the threshold to diagnose metastatic LNs, but many studies have revealed the limitations of using size criteria alone for LN staging in rectal cancer. Approximately 60% of metastatic LNs are < 5 mm in diameter [26]. Therefore, evaluating the shape of the LN can also be useful in diagnosis. In most metastatic LNs, the loss of the fatty hilum and kidney bean-shaped structure can be detected. A recent meta-analysis study including 12 CT studies reported that the pooled sensitivity and specificity of CT for LN metastasis were 71% and 67%, respectively [27]. Another meta-analysis study including 21 MRI studies reported that the pooled sensitivity and specificity of MRI for LN metastasis were 77 and 71%, respectively [28]. In the present study, F-18 FDG PET/CT using a fixed SUV_max_ cut-off value of 2.5 showed low sensitivity (35.8%) and high specificity (97.2%), whereas F-18 FDG PET/CT using SUV_max_ cut-off values optimized according to size showed high sensitivity (76.1%) and high specificity (74.3%). The diagnostic value of the present study is considered to be comparable to those of previous CT or MRI studies. However, direct comparison of diagnostic value is limited between the present study and previous CT or MRI studies, because there are heterogeneities between studies including differences in protocols, radiologists’ experience, approach to image interpretation, and methodologic quality. Further studies with comparison of diagnostic value between F-18 FDG PET/CT, CT, and MRI in the same patient population could provide important information in selecting diagnostic modalities for preoperative staging of rectal cancer.

The limitation of the present study was the use of two different scanners (Discovery STE-16, GE Healthcare; and Biograph mCT-64, Siemens Healthcare), which could not be avoided owing to the retrospective study design. The difference in the resolution and administered dose of F-18 FDG, according to the two different scanners, could have caused differences in the SUV_max_ and could have affected some of the results of the present study. However, previous studies have shown that the difference in the SUV_max_ of the same lesion between two different scanners is < 0.05 [29]. In the present study, there were no significant differences in the SUV_max_ of small and large LNs between the two scanners. Furthermore, the difference between the optimal cut-off values of the two scanners was only 0.1 for small LNs. Although the effect of using two different scanners on the results of the present study would be negligible, further prospective studies involving the use of one PET/CT scanner and a large population are needed for more valid optimal cut-off values.

## Conclusions

Application of the lower cut-off value of SUV_max_ increases the sensitivity of F-18 FDG PET/CT for evaluation of the small regional LNs in patients with rectal cancer. F-18 FDG PET/CT using the optimized SUV_max_ cut-off values according to the LN size has the potential to show improved diagnostic performance for the detection of regional LN metastasis in patients with rectal cancer. Further prospective studies involving the use of one PET/CT scanner and a large population are needed.

## Additional files

Additional file 1: **Table S1.** Comparison of diagnostic values between PET/CT using the optimized cut-off values and the fixed cut-off value of 2.5 in patients with early (T1–2) and advanced (T3–4) T stages. (DOCX 19 kb)

Additional file 2: **Table S2.** Comparison of diagnostic values between PET/CT using the optimized cut-off values and the fixed cut-off value of 2.5 in patients with low (< 13.0) and high (> 13.0) SUV_max_ of primary tumor. (DOCX 17 kb)

## Abbreviations

AUC: areas under the curve
CT: conventional computed tomography
FDG: fluorodeoxyglucose
LN: lymph node
MRI: magnetic resonance imaging
PET/CT: positron emission tomography/computed tomography
ROC: receiver operating characteristic curve
SUV: standardized uptake value
SUV_max_: maximum standardized uptake value
