# Supplementary material for: Accuracy of F-18 FDG PET/CT with optimal cut-offs of maximum standardized uptake value according to size for diagnosis of regional lymph node metastasis in patients with rectal cancer
Source: Cancer Imaging. 2018 Sep 14;18:32. doi: 10.1186/s40644-018-0165-5 (PMC6137872; doi:10.1186/s40644-018-0165-5)
Supplement: Supplementary file 1 — Table S1. Comparison of diagnostic values between PET/CT using the optimized cut-off values and the fixed cut-off value of 2.5 in patients with early (T1–2) and advanced (T3–4) T stages. (DOCX 19 kb) [file 40644_2018_165_MOESM1_ESM.docx]

**Additional file 1**

**Table S1.** Comparison of diagnostic values between PET/CT using the optimized cut-off values and the fixed cut-off value of 2.5 in patients with early (T1-2) and advanced (T3-4) T stages.

| Group | Cut-off values | Sensitivity (%) | Specificity (%) | PPV^*^  (%) | NPV^†^  (%) | Accuracy (%) | AUC^‡^ | *p* |
| --- | --- | --- | --- | --- | --- | --- | --- | --- |
| Early T stage |  |  |  |  |  |  |  |  |
| Overall | 2.5 | 41.7 | 97.1 | 71.4 | 90.5 | 88.9 | 0.694 | 0.298 |
|  | Opt | 100.0 | 62.3 | 31.6 | 100.0 | 67.9 | 0.812 |  |
| Small LN | 2.5 | 22.2 | 100.0 | 100.0 | 89.4 | 89.7 | 0.611 | 0.188 |
|  | 0.9 | 100.0 | 59.3 | 27.3 | 100.0 | 64.7 | 0.797 |  |
| Large LN | 2.5 | 100.0 | 80.0 | 60.0 | 100.0 | 84.6 | 0.900 | 1.000 |
|  | 1.8 | 100.0 | 80.0 | 60.0 | 100.0 | 84.6 | 0.900 |  |
| Advanced T stage |  |  |  |  |  |  |  |  |
| Overall | 2.5 | 34.5 | 97.5 | 95.0 | 52.0 | 61.1 | 0.660 | 0.876 |
|  | Opt | 69.1 | 65.0 | 73.1 | 60.5 | 67.4 | 0.670 |  |
| Small LN | 2.5 | 17.4 | 100.0 | 100.0 | 58.7 | 62.0 | 0.587 | 0.231 |
|  | 1.1 | 91.3 | 51.9 | 61.8 | 87.5 | 70.0 | 0.716 |  |
| Large LN | 2.5 | 46.9 | 92.3 | 93.8 | 41.4 | 60.0 | 0.696 | 0.822 |
|  | 2.1 | 65.6 | 76.9 | 87.5 | 47.6 | 68.9 | 0.713 |  |

^*^PPV: positive predictive value; ^†^ NPV: negative predictive value; ^‡^AUC: area under the curve; [^§^](https://en.wikipedia.org/wiki/Section_sign)Opt: optimal cut-off values of SUV_max_
