# Supplementary material for: Accuracy of F-18 FDG PET/CT with optimal cut-offs of maximum standardized uptake value according to size for diagnosis of regional lymph node metastasis in patients with rectal cancer
Source: Cancer Imaging. 2018 Sep 14;18:32. doi: 10.1186/s40644-018-0165-5 (PMC6137872; doi:10.1186/s40644-018-0165-5)
Supplement: Supplementary file 2 — Table S2. Comparison of diagnostic values between PET/CT using the optimized cut-off values and the fixed cut-off value of 2.5 in patients with low (< 13.0) and high (> 13.0) SUVmax of primary tumor. (DOCX 17 kb) [file 40644_2018_165_MOESM2_ESM.docx]

**Additional file 2**

**Table S2.** Comparison of diagnostic values between PET/CT using the optimized cut-off values and the fixed cut-off value of 2.5 in patients with low (<13.0) and high (>13.0) SUV_max_ of primary tumor

|  | Cut-off values | Sensitivity (%) | Specificity (%) | PPV^*^  (%) | NPV^†^  (%) | Accuracy (%) | AUC^‡^ | *p* |
| --- | --- | --- | --- | --- | --- | --- | --- | --- |
| Low tumor SUV_max_ |  |  |  |  |  |  |  |  |
| Overall | 2.5 | 30.8 | 98.3 | 88.9 | 76.0 | 77.4 | 0.645 | 0.201 |
|  | Opt | 88.5 | 62.1 | 51.1 | 92.3 | 70.2 | 0.753 |  |
| Small LN | 2.5 | 13.3 | 100.0 | 100.0 | 79.0 | 79.7 | 0.567 | 0.070 |
|  | 1.0 | 93.3 | 61.2 | 42.4 | 96.8 | 68.8 | 0.773 |  |
| Large LN | 2.5 | 54.5 | 88.9 | 85.7 | 61.5 | 70.0 | 0.717 | 0.908 |
|  | 1.3 | 90.9 | 55.6 | 71.4 | 83.3 | 75.0 | 0.732 |  |
| High tumor SUV_max_ |  |  |  |  |  |  |  |  |
| Overall | 2.5 | 39.0 | 96.1 | 88.9 | 66.2 | 70.7 | 0.676 | 0.502 |
|  | Opt | 73.2 | 70.6 | 66.7 | 76.6 | 71.7 | 0.719 |  |
| Small LN | 2.5 | 23.5 | 100.0 | 100.0 | 74.0 | 75.9 | 0.618 | 0.177 |
|  | 1.1 | 88.2 | 64.9 | 53.6 | 92.3 | 72.2 | 0.766 |  |
| Large LN | 2.5 | 50.0 | 85.7 | 85.7 | 50.0 | 63.2 | 0.679 | 0.491 |
|  | 2.1 | 75.0 | 71.4 | 81.8 | 62.5 | 73.7 | 0.732 |  |

^*^PPV: positive predictive value; ^†^ NPV: negative predictive value; ^‡^AUC: area under the curve; [^§^](https://en.wikipedia.org/wiki/Section_sign)Opt: optimal cut-off values of SUV_max_
